# Burden of community-acquired and nosocomial rotavirus gastroenteritis in the pediatric population of Western Europe: a scoping review

**DOI:** 10.1186/1471-2334-12-62

**Published:** 2012-03-19

**Authors:** Isla Ogilvie, Hanane Khoury, Mireille M Goetghebeur, Antoine C El Khoury, Carlo Giaquinto

**Affiliations:** 1 BioMedCom Consultants Inc., 1405 TransCanada Highway, Suite 310, Montreal, QC, H9P 2V9, Canada; 2 Merck & Co, West Point, PA 19486, USA; 3 Department of Paediatrics, University of Padua, Padua, Italy

**Keywords:** Rotavirus, Burden of illness, Gastroenteritis, Pediatric population

## Abstract

**Background:**

Rotavirus affects 95% of children worldwide by age 5 years and is the leading cause of severe dehydrating diarrhea. The objective of this review was to estimate the burden of rotavirus gastroenteritis (RVGE) in the Western European pediatric population.

**Methods:**

A comprehensive literature search (1999-2010) was conducted in PubMed and other sources (CDC; WHO, others). Data on the epidemiology and burden of RVGE among children < 5 years-old in Western Europe --including hospital-acquired disease--were extracted.

**Results:**

76 studies from 16 countries were identified. The mean percentage of acute gastroenteritis (AGE) cases caused by rotavirus ranged from 25.3%-63.5% in children < 5 years of age, peaking during winter. Incidence rates of RVGE ranged from 1.33-4.96 cases/100 person- years. Hospitalization rates for RVGE ranged from 7% to 81% among infected children, depending on the country. Nosocomial RVGE accounted for 47%-69% of all hospital-acquired AGE and prolonged hospital stays by 4-12 days. Each year, RVGE incurred $0.54- $53.6 million in direct medical costs and $1.7-$22.4 million in indirect costs in the 16 countries studied. Full serotyping data was available for 8 countries. G1P[8], G2P[4], G9P[8], and G3P[8] were the most prevalent serotypes (cumulative frequency: 57.2%- 98.7%). Serotype distribution in nosocomial RVGE was similar.

**Conclusions:**

This review confirms that RVGE is a common disease associated with significant morbidity and costs across Western Europe. A vaccine protecting against multiple serotypes may decrease the epidemiological and cost burden of RVGE in Western Europe.

## Background

Rotavirus affects 95% of all non-vaccinated children worldwide by the age of five years, and is the leading cause of severe dehydrating diarrhea in that age group [1,2]. Among the 23.6 million children under five years of age in the European Union, it is estimated that 3.6 million episodes of rotavirus gastroenteritis (RVGE) occur annually [3]. Transmitted by the fecal-oral route, most rotavirus infections are community-acquired, however nosocomial infections are a major component of hospital-acquired infections in children [4]. In a review from six countries in Europe in 2006, RVGE was reported as the major etiologic agent of pediatric nosocomial diarrhea, accounting for 31-87% of cases [4].

Rotavirus infection peaks in the winter months between November and February in temperate climates [1,4,5]. The predominance of a particular rotavirus genotype combination during an RVGE season may vary between geographical areas, and from one season to the next [6]. Of the seven sero-groups of rotavirus (groups A-G), three are capable of affecting humans (groups A-C). Group A rotavirus infections are the most common [5]; they are further classified into G- and P-types [7]. Rotavirus carrying either G1, G2, G3, G4, or G9 genotypes combined with P[4] or P[8] genotypes are currently the most common causes of RVGE in humans representing more than 90% of all RVGE cases observed in Europe, with G12 an emergent genotype [8].

Within the European Union, RVGE is estimated to occur at a rate of 1 symptomatic infection in every 7 children each year, accounting for 231 deaths, more than 87,000 hospitalizations and almost 700,000 outpatient visits [3]. Many episodes of RVGE are mild enough to be managed at home, however, within Europe, approximately 20% of children will see a medical practitioner during the illness, and an estimated 1 in 54 cases will result in hospitalization [3]. RVGE imposes a heavy economic burden by incurring not only direct (consultation, emergency, hospitalization, medication) costs, but also indirect costs (parent work days lost, childcare, etc....) [3,9,10].

### Objectives of study

The objective of this study was to carry out a comprehensive scoping review of the recent literature on the burden of pediatric RVGE, both nosocomial and community-acquired, in Western Europe. We examined the epidemiology of RVGE, the distribution of rotavirus genotype combinations, morbidity and mortality due to RVGE, resources utilization and costs by country and according to healthcare setting.

## Methods

### Literature search strategy

A comprehensive literature search was conducted for studies pertaining to the burden of rotavirus infection on the pediatric population in Western Europe (< 5 years, unless otherwise specified). The searches, limited to articles published between 1^st ^January 1999 and 1^st ^May 2010, were carried out in the National Library of Medicine's Pubmed, the Center for Disease Control (CDC) rotavirus global surveillance http://www.cdc.gov/rotavirus/global_surveillance/surveillance.htm, and the World Health Organization (WHO) http://www.who.int/nuvi/rotavirus/en/. Studies were included if they were set in the following countries: Austria, Belgium, Denmark, Finland, France, Germany, Greece, Ireland, Italy, the Netherlands, Norway, Portugal, Spain, Sweden, Switzerland and the United Kingdom (UK). The search terms used for this scoping review included: rotavirus, outcome, mortality, death, incidence, prevalence, nosocomial, home care, serotype, strain, cost, economic, burden, and resource use, where the asterisk represents a wildcard. English language articles were primarily reviewed along with promising articles in other languages. In addition, records were searched manually to identify the most relevant studies pertaining to the object of this document and bibliographies of retrieved articles were screened to identify additional sources of information.

### Data extraction and analysis

For all studies, the dates reported refer to when studies were conducted, which was often several years before the publication date. Where data was available for an individual country within a multicountry study, both overall and data delineated by country were extracted. Where available, information was reported for both community-acquired and nosocomial RVGE, and from hospital, primary care or home care settings. An RVGE episode was considered to be nosocomial (hospital-acquired) if gastroenteritis symptoms occurred at least 48 h after children were admitted to hospital for a diagnosis other than diarrhea [4].

### Epidemiology of RVGE

Incidence data was extracted as cases of RVGE per 100 patient-years for countries of interest. Information on incidence from studies conducted in a day-care setting and for nosocomial infection was captured separately. Where incidence data was reported as per child month, the original data were reported. In addition data was converted to person-years, for ease of comparison between settings. Likewise, incidence of nosocomial disease reported as cases per 1000 days of hospitalization was also provided as cases per 100 person-years in hospital.

The proportion of RVGE among cases of acute gastroenteritis was extracted. In the case where several surveillance studies were published for a single country, a pooled average of the proportion of RVGE among cases of acute gastroenteritis was calculated and reported, along with ranges across studies for each country. If available, the variation over time in the proportion of RVGE among cases of gastroenteritis was captured.

Data on infection seasonality was collected and reported.

### Rotavirus genotype combinations

The proportion of each genotype combination among genotyped RVGE samples was extracted, where available. The most recently available studies from each country were used to gather information about the distribution of genotype combinations across the countries of interest. In addition, data concerning changes in the prevalence of genotype combinations over time was collected, as was any reference to emerging serotypes.

### Morbidity and mortality

To assess disease severity, two types of data were extracted, where available. Firstly, the severity and proportion of patients suffering from dehydration due to RVGE was captured and secondly, disease severity as measured by the 20-point Vesikari scoring system [11]. The Vesikari scale is based on the duration and intensity of diarrhea and vomiting, intensity of fever and dehydration, and need for treatment and hospitalization [11]. A Vesikari score ≥ 11 is indicative of severe disease [11].

Mortality due to RVGE was captured as annual fatalities and mortality rates per 100,000 children less than five years of age.

### Disease burden

For healthcare resource utilization, data on hospital admission rates, need for intravenous rehydration, and duration of hospital stay were collected. Hospitalization due to RVGE was defined as hospital admission of children presenting to hospital with severe acute community-acquired RVGE [12]. Cost-of-illness data captured included direct medical costs, out-of-pocket expenditures, and indirect costs attributed to lost productivity by parents of children suffering from RVGE. Costs are reported in 2009 US dollars (1 $US equals 0.721189 € [1^st ^Jan 2009]) [13]. In addition, where a national cost estimate for the total cost of RVGE was available, we have presented the total cost of illness as a proportion of the gross national product of that country, to enable comparison between countries. The gross national product for each country for 2009 was obtained from the World Bank at http://data.worldbank.org/data-catalog[14].

## Results

### Literature review

#### Study selection

**Figure 1:**
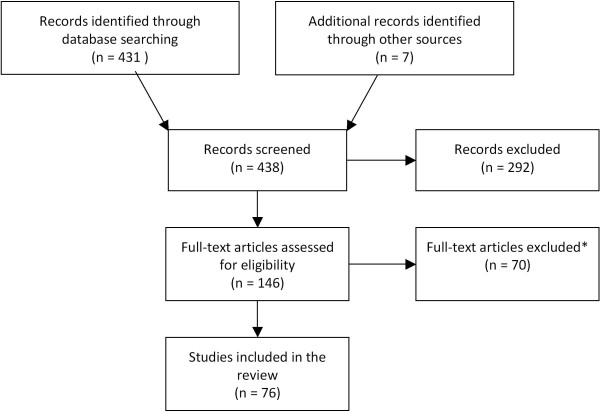
**PRISMA diagram of studies selected**.

As shown in Figure 1, a total of 76 studies containing relevant data pertaining to RVGE in children under five years of age were captured. Studies were included on the following topics: incidence (n = 16 studies), proportion of RVGE among cases of acute gastroenteritis (n = 38 studies), seasonality of RVGE infection (n = 36 studies), serotype distribution (n = 25), disease severity (n = 20), mortality (n = 9), healthcare resource utilization (n = 36), and costs (n = 17). More than one type of data was available from a single study; hence the total number of studies is not the sum of those for each topic. A study was excluded if it was not carried out in humans, was a duplicate of what was already included, was not from a country of interest, did not include children under five years of age, if there were no original data (i.e. a review), or if data did not meet the criteria given above.

#### Overview of evidence available

An overview of the evidence available for each country is presented in Table 1, and detail about each study selected is given in the Additional file 1. Epidemiological data for RVGE in Western Europe was available from a total of 52 studies covering incidence, the proportion of RVGE among acute gastroenteritis cases, and seasonality of infection.

**Table 1:** Literature capture and data sources

Country	Epidemiology			Genotype combination data	Morbidity and Mortality		Disease Burden	
	Incidence	Proportion RVGE	Seasonality		Disease severity	Mortality	Resource utilization	Costs
Multic ountry*	15**,1 6^¶^, 25^║^	25^║^,34^¶,^85^§^	15**,16^¶^,25^║^,36^‡^	15**,25^║^,3 6^‡^,92^†^,93^¶^	15**,25^║^,34^¶^	78^††^	15**,25^║^,34^¶^,85^§^	83^¶^
Austria	15**,31	31	15**,31	62	31,76	78^††^		76
Belgium	16^¶^	34^¶^	16^¶^	93^¶^,94	81	78^††^	81,85^§^	81,83^¶^
Denmark			45,96	36^‡^,12	95	78^††^	45, 85^§^,95,96	45
Finland			23			78^††^	23,76^§^	
France	16^¶^,17,20,21,25^║^,26,27,28	17,25^║^,34^¶^,26,27,28,37,38,40,41,49,97,98	16^¶^,17,20,21,25^║^,26,27,36^‡^,39,40,41,98	17,20,25^║^,36^‡^,63,93^¶^	17,20,26,37,38,49,79	17,26,78^††^	17,20,21,26,27,34^¶^,37,38,3 9,49,79,85^§^,87	20,21,27,38,74^¶^,87,98
Germany	15**,1 6^¶^,18,25^║^	25^║^,34^¶^,42,50	15**,16^¶^,18,25^║^,42	25^║^,50,93^¶^		42,78^††^,9 9	34^¶^,42,85^§^	83^¶^
Greece		46,47	46,47		46	78^††^	46,85^§^	
Ireland			44		77	78^††^	44,77,85^§^	77
Italy	16^¶^,19, 25^║^,32	19,25^║^,32,34^¶^,51,52, 53,100	16^¶^,19,25^║^,3 2,36^‡^,51,100	19,25^║^,51, 60,65,68,9 3^¶^	19,51, 82		19,32,34^¶^,52,82,85^§^	32,82, 83^¶^
Netherl ands			101			78^††^	85§	
Norway		102,103	103	102		78^††^		
Portugal		61,104	104	61,104		78^††^		
Scandinavia			36^†^					
Spain	16^¶^,25^║^,29,30,33	25^║^,29,30,32,34^¶^,54,55,56	16^¶^,25^║^,29,30,33,36^‡^,54,90,105	25^║^,30,36^‡^, 55,64,93^¶^		29,3 0,56,78^††^,	29,30,34^¶^,56,85^§^,89, 90,105	29,56,83^¶^,89, 90
Sweden	16^¶^	34^¶^	16^¶^	93^¶^	43	78^††^	34^¶^,85^§^	83^¶^
Switzerland	15**		15**					
UK	16^¶^,25^║^	34^¶^,57,58,59	16^¶^,25^║^,36^‡^,57	25^║^,36^‡^,57,58,93^¶^		78^††^,106	34^¶^,85^§^,91, 106	83^¶^,91

*Where data was available for individual countries within a multicountry study, it has been extracted. Therefore, the relevant reference has been added to individual countries above

^†^Denmark, Finland, Germany, Hungary, Italy, Netherlands, Slovenia, Spain, Sweden, Bulgaria, Lithuania, Belgium, UK

^‡^Denmark, France, Italy, Scandinavia, Spain, UK

^§^Belgium, Denmark, Finland, France, Germany, Greece, Ireland, Italy, The Netherlands, Spain, Sweden, UK, Overall data WHO European region

^║^Western Europe, France, Germany, Italy, Spain, UK

^¶^Belgium, France, Germany, Italy, Spain, Sweden, UK

**Austria, Switzerland and Germany

^††^Austria, Belgium, Denmark, Finland, France, Germany, Greece, Ireland, Italy, The Netherlands, Norway, Portugal, Spain, Sweden, UK

### Epidemiology of RVGE

#### Incidence of community-acquired RVGE

The annual incidence of community-acquired RVGE among children under five years of age (per 100 person years) was reported in seven countries [15-19], ranging from 1.33 cases per 100 person-years (in Austria) to 4.96 cases per 100 person-years (in France) [15-19]. The incidence of RVGE was approximately 12-fold higher among children under three years of age in a daycare setting--between 2.2 and 2.5 cases per 100 child-months (26.4-30 cases per 100 person-years)--than for children in community-based studies [20,21].

##### Home care setting

A high number of children with RVGE are not sick enough to be admitted to hospital and many patients receive no medical care [22]. Since rotavirus infection is not a notifiable disease and the exact diagnosis is not needed for individual patient management, the incidence rates given above may be an underestimate of the total disease incidence, and refer only to patients requiring medical attention. Examination of the literature showed that data concerning RVGE patients treated at home is sparse, most studies estimated the proportion of patients with RVGE receiving home care. For Europe, estimations of the proportion of RVGE patients receiving no medical care ranged from 25% to 51% of patients [3,23,24]. Two studies from a day care setting in France reported that 34.6% and 14.3% of RVGE cases, respectively, did not seek medical attention [20,21].

#### Incidence of nosocomial RVGE

The incidence of nosocomial infection with RVGE reported among the hospitalized pediatric population was also higher than the incidence reported in community based studies [25]. A prospective study by Forster et al. that examined the incidence of RVGE among patients under five years old in hospitals from five countries (France, Germany, Italy, Spain and the UK) reported an overall incidence of nosocomial RVGE of 0.46 per 1000 days of hospitalization (16.8 per 100 person-years in hospital) [25]. The range across countries varied from 0 to 1.87 cases per 1000 days of hospitalization (0-68.2 per 100 person-years in hospital) [25]. Other studies from Austria, France, Germany, Italy, Spain, and Switzerland reported incidence rates for nosocomial RVGE within the range reported by the Forster study [15,26-33].

#### Proportion of community-acquired RVGE

**Figure 2:**
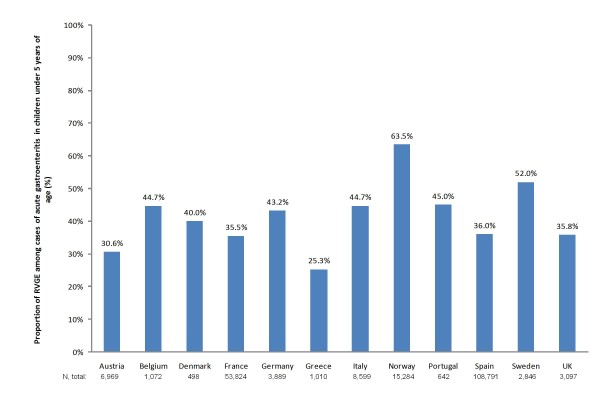
**Mean overall proportion of rotavirus infection among reported acute gastroenteritis cases by country**.

When limited to those studies that reported the proportion of community-acquired RVGE among reported gastroenteritis cases for children under five years of age, the mean percentage of cases caused by rotavirus infection ranged from 25.3% to 63.5%. Greece reported the lowest proportion (25.3%), while those with the highest proportion included Norway (63.5%) and Sweden (52.0%). The remainder of the countries reported a percentage of between 36% and 45% (Figure 2). These rates vary greatly depending on whether patients are seen in hospital, emergency room, or primary care physician clinic.

The REVEAL study, carried out in Belgium, France, Germany, Italy, Spain, Sweden, and the UK, reported that RVGE in children under five years of age was responsible for between 53.0% and 68.9% of cases presenting to hospitals, 35.4% and 63.3% for those seen in emergency departments, and 7.7% and 41.3% of cases seeking primary care physicians [34]. A second prospective multicenter study of children under five years of age from France, Germany, Italy, Spain and the UK reported that the overall proportion of community-acquired rotavirus infections among reported hospitalized and ED acute gastroenteritis cases across the five countries was 43.4% [25]. Most of these cases (80.9%) occurred in children under 2 years of age [25]. In the REVEAL study, 86.1% of RVGE cases occurred in children aged between 3 months and 3 years of age while a larger study from EuroRotaNet reported that 81.5% of cases from 19 European countries were in patients under 2.5 years of age [35,36].

#### Proportion of nosocomial RVGE

Only a few methodologically weak studies were identified relating to the prevalence of nosocomial RVGE among children hospitalized for other conditions, or the proportion of acute nosocomial gastroenteritis that was due to rotavirus. In the original reports, nosocomial RVGE was generally defined as disease occurring between 48 to 72 h after admission of the child to hospital, depending on the study [15,26,37]. The prevalence of nosocomial rotavirus infections among hospitalized children admitted for a diagnosis other than diarrhea ranged from 2.9% to 6.6% in four studies from France [26,27,37,38]. Three studies from five countries (Austria, France, Germany, Spain and Switzerland) examined the proportion of nosocomial RVGE among cases of acute nosocomial gastroenteritis [15,26,30]. Nosocomial RVGE accounted for between 47% and 69% of all hospital-acquired acute gastroenteritis among hospitalized children in the studies covering Austria, Germany, Spain and Switzerland [15,30]. In one French study conducted in 1999 during the peak of the rotavirus season, 97.8% of nosocomial diarrhea cases were due to rotavirus [26]. A comparison of community-acquired RVGE and nosocomial RVGE was carried out in a study from Austria, Germany and Switzerland [15]. Rotavirus was detected in 29.5%, 27% and 37.5% of children with community-acquired gastroenteritis, and in 57%, 69% and 49% of children with nosocomial gastroenteritis, in Austria, Germany and Switzerland, respectively [15]. The seasonality of nosocomial RVGE mirrored that of RVGE in the broader community, with most infections taking place in the winter months [15,25-27,29,30,39-42].

Patients with nosocomial disease were generally younger than those who acquired RVGE in the community [15,26,29,42,43]. Between 42.7% and 69.9% of children affected by nosocomial RVGE were under 6 months of age (mainly neonates) compared with 15.9% to 33.6% of patients with community-acquired disease [25,42]. Across three studies covering five countries (Austria, France, Switzerland, Germany and Sweden), the median age of patients with nosocomial RVGE was between 2.8 and 9 months compared to a median age of between 12.5 and 16.7 months for patients with community-acquired RVGE [15,39,43]. Four risk factors were reported to be strongly associated with nosocomial RVGE in neonatal intensive care units including premature birth (Odds Ratio [OR]: 2.63; *P *< 0.001), infections other than rotavirus (OR: 2.35; *P *< 0.01), malformation (OR: 2.38; *P *< 0.01), and changes in glycemia and/or presence of jaundice (OR: 7.63; *P *< 0.001) [33].

#### Seasonality of RVGE

For most countries in Western Europe, RVGE was reported to occur most often during the winter and spring, from December to April or May. A slightly later rotavirus season was noted in Ireland (February to June), Greece (January to April), and Scandinavian countries (Denmark: January to June; Norway: March to May; Sweden: April) [23,36,44-48] No clear trend was found in relation to geographical or climatological parameters.

### Rotavirus genotype combinations

#### Distribution of genotype combinations

##### Community-acquired RVGE

The predominant serotype of rotavirus causing RVGE varies from country to country and from year to year. The most recent serotype data from each country is presented here. Only one genotype combination (G1P[8]) was reported in all eight countries for which full serotyping data is available (Austria, Denmark, France, Germany, Italy, Portugal, Spain and the UK). G1P[8] was the most common genotype combination in Austria [15], Denmark [36]. France [36], Germany [25], Spain [25,36], and the UK [36], accounting for between 48.6% and 84.7% of genotyped RVGE samples in those countries.

##### Nosocomial RVGE

The distribution of rotavirus genotype combinations among patients with nosocomial RVGE was reported in four studies covering seven countries (Austria, France, Germany, Italy, Spain Switzerland and the UK) [15,25,30,49]. In general, the distribution of rotavirus genotype combinations among patients with nosocomial RVGE was very similar to the distribution among patients with community-acquired disease from the same study [15,25,30,49]. For example, in a prospective multicenter study from France, Germany, Italy, Spain and the UK, G1P[8] accounted for 40.3% of genotyped samples among patients with community-acquired RVGE and 35% of nosocomial RVGE [25]. Similarly in the same study, G9P[8] accounted for 31.2% of community-acquired RVGE and 36.3% of nosocomial RVGE, and the proportions of other genotype combinations were similar [25].

#### Evolution over time of genotype combinations

##### Community-acquired RVGE

**Figure 3:**
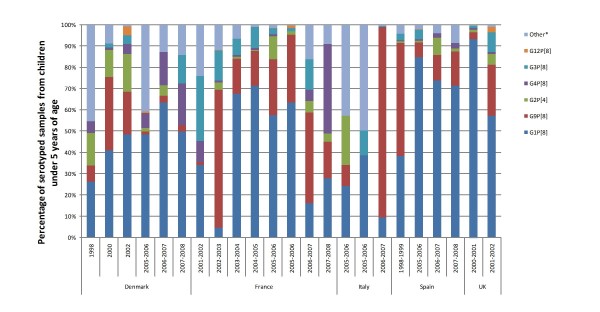
**Distribution of genotype combinations in Western Europe over time, from recent literature**. *Other includes rare genotype combinations, mixed genotype combinations and non-typable and partially typable samples.

Information concerning serotype distribution over time for fully serotyped samples from children under five years of age was available for five countries (Denmark [36,50], France [25,36,51], Italy [25,52,53], Spain [25,30,36,54], and the UK [25,36,55,56]) (Figure 3). However, on examination of the evolution of genotype distribution and predominance over time, we were not able to discern any overall trends in serotype distribution within the region. This is because serotype predominance appears to change on a season to season basis within each country, and may even differ from region to region within the same country.

##### Nosocomial RVGE

No studies examined the distribution of genotype combinations among patients with nosocomial RVGE over time.

#### Emerging rotavirus serotypes in Western Europe

Four studies, three from Italy and one from Spain described the emergence of new rotavirus serotypes in Western Europe [52,54,57,58]. The emergence of the G9 serotype was documented in the winter season of 1999-2000 in Southern Italy (19% of all rotaviruses) [59], becoming then most prevalent in other European regions An earlier study from Barcelona, Spain, documented an increase in the levels of strains G1P[4] and G3P[4], which were considered uncommon at the time of the study (1998-1999). Additionally, two G5 strains were isolated in Barcelona; the origin of these G5 strains (porcine or human) was unclear [54]. G12P[8], an emerging potential human pathogen,[8] was documented in Denmark in 2002 (4.1% of all rotaviruses) and the UK (2.4%),[36] and was detected in very small proportions (< 1%) in France[36,51] and Spain [36].

### Morbidity and mortality

#### Disease severity

##### Community-acquired RVGE

Disease severity assessment using the 20-point Vesikari scale was reported by seven studies covering eight countries [15,17,20,25,31,46]. In France and Greece, the proportion of children infected by rotavirus having moderate to severe gastroenteritis was significantly higher compared to children with non-rotavirus infection (82.8% vs 37.2% [*P *< .0001] in France; 65.4% vs 41.2% [*P *< .01] in Greece) [17,46]. The proportion of patients presenting to hospitals with severe acute community-acquired RVGE (Vesikari score ≥ 11) was between 29% and 55% in a study covering Austria, Germany, and Switzerland; a study set in France, Italy, Spain and the UK reported an overall proportion of severe disease of 53.3% [15,25].

##### Nosocomial RVGE

For patients with hospital-acquired infection, the overall proportion of patients with severe RVGE in France, Italy, Spain and the UK was 42.6%, while for Austria, Germany, and Switzerland severe infection affected 24.4%, 30.2% and 40%, respectively [15,25].

##### Community-acquired versus nosocomial RVGE

In a study from Austria, Germany, and Switzerland, overall disease severity scores, by country, were similar in community-acquired (median score: 9-11) and nosocomial RVGE (median score: 8-9) [15]. When community-acquired RVGE and non-RVGE were compared, disease severity scores were significantly higher for rotavirus-positive gastroenteritis with a median score of 11 in Austria (vs 7 for non-rotavirus; *P *< .001) [31], and a mean score of 12.14 in France (vs 6.93 for non-rotavirus, *P *< .0001) [20].

##### Dehydration in community-acquired RVGE

The REVEAL study reported that the proportion of children with dehydration due to acute RVGE varied between 11.1% (Spain) and 71.4% (Sweden), and in most countries, was considerably higher than for those with rotavirus-negative disease [34]. In Belgium and the UK, the prevalence of dehydration was comparable among children with or without RVGE [34], whereas the dehydration prevalence ratio of rotavirus versus non-rotavirus disease was 1.82, 5.54, 3.27, 3.47, and 2.18, respectively, in France, Germany, Italy, Spain, and Sweden [34]. A second prospective multicenter study from France, Germany, Italy, Spain, and the UK reported that dehydration was evident in 75.7% of patients with RVGE, and was severe in 11.3% of them [25]. In comparison, only 54.5% of children with non-RVGE were dehydrated, while 4.7% had severe dehydration [25]. Another study from France confirmed this trend, showing significant differences in dehydration between rotavirus-positive and negative gastroenteritis (26.8% vs.14.7%, *P *< .0001) as did a study from Greece [17,46]. Further, one Italian community-based study showed that dehydration at initial presentation in primary care was associated with a higher likelihood of RVGE (OR: 1.8; 95% CI, 1.1-3; *P *= 0.02) [53].

##### Dehydration in community-acquired versus nosocomial RVGE

Only two studies compared the proportion of patients with dehydration for community-acquired RVGE with those for nosocomial infection; in both studies more patients with community-acquired disease experienced dehydration [43,60]. In an Irish study, 80% of patients with community-acquired disease were dehydrated versus only 55% with nosocomial disease [60]. Likewise, in a study from Sweden, 10.8% of patients with community-acquired RVGE had either hypertonic dehydration, or severe dehydration needing intensive care treatment compared to only 0.8% of patients with nosocomial RVGE [43].

#### Mortality

Rotavirus fatalities were rare among young children in Western Europe with less than 10 deaths occurring per year in most countries [61], but appeared to pose a greater risk for children of younger age. For example, mortality due to nosocomial RVGE was highest among children less than 12 months of age compared to older ones (0.74 per 100,000 vs 0.16 per 100,000 children < 5 years per year), as suggested by a study from Spain [29].

### Disease burden

#### Resource utilization

##### Community-acquired RVGE

In the REVEAL study, the proportions of hospital and emergency referrals among children presenting at primary care with acute RVGE ranged from 13.0% (relative risk [RR]: 3.37; 95% confidence interval [CI]: 1.77-6.43) to 57.1% (RR: 2.10; 95% CI: 0.92-4.75), and from 6.1% to 45.3% (RR: 2.80; 95% CI: 1.68-4.67), respectively, for all countries included in the study [34]. Additional country-specific studies reporting hospital admission rates for community-acquired disease due to acute RVGE were retrieved (France 81% [62]; Germany 7% [15]; Italy 11.2% [19]; Spain 9.2% [12]). In Greece, hospital admissions due to RVGE were significantly more frequent than non-RVGE (51.4% vs 22% non-rotavirus; *P *< 0.01) [46].

In the REVEAL study, hospital stay due to acute RVGE ranged from 2.5 days (Sweden) to 5.0 days in Germany [34]. In Denmark, 63% of young children hospitalised for rotavirus associated disease stayed for 3 days or less [45]. In France, hospitalization due to RVGE was longer than for other viral gastroenteritis cases [63].

##### Nosocomial RVGE

As reported in several studies, nosocomial rotavirus infection prolonged hospital stay, by 4.4 days in Belgium and Italy (national registry data) [64,65], and between 9 and 12 days in Ireland [44,60]. In France, nosocomial RVGE caused statistically significant prolongation of hospital stay, as reported in a prospective observational multicentre study (10 vs 3.9 days; *P *< .001) [26].

#### Costs

Cost of illness and productivity loss data due to RVGE was available from several studies in Western Europe (Table 2).

**Table 2:** Costs associated with rotavirus acute gastroenteritis in Western Europe, 2009US$

Country/Study Costs per patient							National cost estimate per year (per GDP)	
	Direct costs			Indirect costs			Direct costs	Indirect costs
	Hospital	Primary care	Home care	Hospital	Primary care	Home care		
**Austria**								
Fruhwirth, 2001[66]	384 *NRV: *3,752	NA	NA	NA	NA	NA	NA	NA
**Belgium**								
Bilcke, 2009[67]	NA	NA	22	NA	NA	NA	10,258,095 (22)	15,372,592 (33)
Bilcke, 2008[64]	NA	NA	24	NA	NA	253	10,832,292 (23)	22,448,225 (48)
Giaquinto, 2007[68]	2,109	110	NA	287	623	NA	NA	NA
**Denmark**								
Fischer, 2007[45]	NA	NA	NA	NA	NA	NA	2,211,901 (7)	NA
**France**								
Fau, 2008[20]	44*		NA	271*		NA	NA	NA
Melliez, 2008[24]	1,613	53	10	NA	NA	NA	33,829,068 (13)	NA
Giaquinto, 2007[68]	2,103	121	NA	260	377	NA	NA	NA
Huet, 2007[69]	NA	NA	NA	NA	NA	NA	152,230,808 (57)^†^	
Melliez, 2005[70]	NA	NA	NA	NA	NA	NA	35,003,757 (13)	NA
Sermet Gaudelus, 2004[71]	*NRV: *7,185	NA	NA	NA	NA	NA	NA	NA
Piednoir, 2003[27]	*NRV: *4,534	NA	NA	NA	NA	NA	NA	NA
**Germany**								
Giaquinto, 2007[68]	2,306	146	NA	925	524	NA	NA	NA
**Ireland**								
Harrington, 2003[60]	1,428 *NRV: *7,856	NA	NA	NA	NA	NA	NA	NA
**Italy**								
Marsella, 2009[72]	2,075-2,945	NA	NA	NA	NA	NA	NA	NA
Panatto, 2009[65]	2,076	26	NA	NA	NA	NA	543,775 (0.26)	1,763,070 (0.83)
Giaquinto, 2007[68]	1,982	81	NA	964	372	NA	NA	NA
**Spain**								
Gil-Prieto, 2009[29]	4,880	NA	NA	NA	NA	NA	53,643,376 (37)	NA
Lopez-de- Andres, 2008[73]	1,905	NA	NA	NA	NA	NA	8,967,305 (6)	NA
Luquero Alcalde, 2008[74]	4,505	NA	NA	NA	NA	NA	NA	NA
Giaquinto, 2007[68]	1,973	64	NA	432	194	NA	NA	NA
Gil, 2004[75]	1,838	NA	NA	NA	NA	NA	NA	NA
**Sweden**								
Giaquinto, 2007[68]	2,389	260	NA	866	716	NA	NA	NA
**UK**								
Lorgelly, 2008[76]	113 ± 203	NA	NA	157 ± 283	NA	NA	NA	NA
Giaquinto, 2007[68]	1,942	141	NA	1,061	440	NA	NA	NA

NRV: nosocomial rotavirus

*pooled data from hospital & primary care settings

^†^total including direct and indirect

NB: Indirect costs include work days lost by parents of children hospitalized for RVGE and out-of-pocket expenses; direct medical costs include hospitalization, outpatient consultation, prescribed & over the counter medication, and medical services (diagnostic, lab tests, etc....)

##### Hospital setting

In the REVEAL study, acute RVGE was associated with direct medical costs per patient ranging from $1,942 (UK) to $2,389 (Sweden) (Table 2) [68]. In the same setting, indirect costs including work days lost by parents of children hospitalized for RVGE as well as out-of-pocket expenses ranged between $260 (France) and $1,061 (UK) [68]. A portion of indirect costs was attributed to work days lost by parents per hospitalization episode, which varied between 2.3 days (France) and 6.4 days (Germany) [68].

##### Primary care setting

Indirect costs attributed to out-of-pocket expenses and work days lost by parents of children with RVGE ranged between $194 (Spain) and $623 (Belgium) (Table 2) [68]. In this setting, work days lost by parents per episode of RVGE varied between 3.4 days (France) and 7.5 days (UK) [68].

##### Home care setting

Cost data of RVGE management among children who do not seek medical care is limited (Table 2) [21,24,64,67]. In France and Belgium, direct medical costs relating to the routine medical care of RVGE patients (over-the-counter medication) varied between $10 and $24 per patient [24,64,67]. A prospective study estimated the cost of care of children affected by RVGE while attending day care centers at $27; this estimate included the cost of over-the-counter medication, extra diapers, and parent work days lost [21]. Finally, in Belgium, an economic analysis estimated at $937,231 the annual burden of rotavirus disease among children less than 7 years of age in the home care setting [67].

##### National cost estimates

In Western Europe, rotavirus disease incurred direct medical costs of $543,775 (Italy) [65] to $53.6 million (Spain) [29] each year, and between $1.7 million (Italy) [65] and $22.4 million (Belgium) [64] in indirect costs (Table 2).

## Discussion

Based on the currently available literature on rotavirus burden in Western Europe, this analysis revealed that incidence rates for RVGE in the under five year old population ranged between 1.33 and 4.96 cases per 100 person-years [15-19]. However, the incidence rate of RVGE is likely underestimated as many patients receive care at home. Estimation of the proportion of RVGE patients receiving no medical care ranged from 25% to 51% of patients [3,23,24]. Accurate estimates of total incidence rates accounting for all these RVGE cases were not possible due to the limited availability of epidemiological data from community-based cohort studies. The incidence of nosocomial infection due to RVGE reported among the hospitalized pediatric population is higher than the incidence reported in community-based studies ranging from 0 to 1.87 cases per 1000 days of hospitalization (0-68.2 per 100 person-years in hospital) depending on the country [25].

The prevalence of rotavirus infection varied from country to country. Among the pediatric population, community-acquired rotavirus infection accounts for between 25.3% (in Greece) to 63.5% (in Sweden) of acute gastroenteritis cases [77-79]. These variations may reflect actual differences in the proportion of RVGE and incidence of rotaviral disease, however, variations in the design of the studies captured in the review limits comparability across countries. Nosocomial RVGE accounted for between 47% and 69% of all hospital-acquired acute gastroenteritis among hospitalized children except in one study, conducted in France during the peak of the rotavirus season, where 97% of nosocomial diarrhea cases were due to rotavirus [15,26,30]. For most countries in Western Europe, the season for RVGE was reported to occur in the winter from December to April or May. A slightly later rotavirus season was noted in Ireland, Greece and Scandinavian countries. Nosocomial infection mirrored the seasonality of the community-acquired disease.

The most commonly isolated genotype combinations in the Western European region were G1P[8], G2P[4], and G9P[8] according to the most recent studies available for each country. G2P[8] and G3P[8] were also widespread in the region. Genotype combinations G1P[4], G4P[9], G9P[9], G9P[4], and G2P[10] were each reported in only one country. Non-typable and partially typed serotypes accounted for between 0.0% and 3.8% of all serotypes in studies where these were reported. In general, the distribution of rotavirus genotype combinations among patients with nosocomial RVGE was very similar to the distribution among patients with community-acquired disease from the same study [15,25,30,49].

While several studies tracked the evolution of genotype distribution and predominance over time, we were not able to discern any overall trends in serotype distribution within the region. This is because serotype predominance appears to change on a season to season basis within each country, and may even differ from region to region within the same country. Emerging rotavirus serotypes were rarely reported in Italy and Spain.

Rotavirus fatalities were rare across the region with less than 10 deaths occurring per year in most countries among children under five years old; therefore, few studies included mortality data for RVGE. Mortality due to nosocomial RVGE was higher, reaching 0.74 per 100,000 children-year among children of less than 12 months of age compared to 0.16 per 100,000 children-year for those < 5 years) [29]. Comparison with a global literature review of mortality due to rotavirus shows that all of the countries in Western Europe have some of the lowest mortality rates globally [22]. A recent literature review of rotavirus burden of illness in the Middle East and North Africa reported annual mortality rates of between 0 and 112 per 100,000 children under five years of age depending on the country [80].

Resource utilization by rotavirus infected patients is generally higher than for non-RVGE related disease. Hospital admission for gastroenteritis is significantly more likely to happen in RV induced infections than for non-RV related disease [46] and, when compared to non-RVGE, RVGE is associated with significantly higher disease severity scores [20,31]. Intravenous rehydration was more commonly administered to patients with acute RVGE in an emergency department or hospital setting, compared to patients with non-rotavirus disease, reflecting the higher level of dehydration in RVGE patients [25,34]. In addition, hospitalization due to RVGE was longer than for other viral gastroenteritis causes [63].

Overall, the duration of hospital stay ranged between 2.5 days to 5.0 days for patients with community-acquired RVGE while nosocomial RVGE infection prolonged hospital stay by 4.4 days [68]. However, similar disease severity scores were reported for community-acquired and nosocomial RVGE. Little data is available concerning dehydration in nosocomial cases.

The cost of illness and productivity loss due to RVGE is large. Overall, at the country level, direct medical costs due to RVGE ranged between $543,775 and $53.6 million according to the size of the pediatric population and the type of health care provided, while indirect costs accounted for an additional $1.7 million to $22.4 million, annually [29,64,65]. Cost per patient varied by setting, with patients hospitalized for RVGE incurring the highest direct and indirect costs, and patients treated at home incurring the lowest costs; however, data pertaining to the cost of RVGE management among children who do not seek medical care is limited [21,24,64,67,68].

## Limitations

Study limitations include the lack of available information on the burden of RVGE in terms of mortality, nosocomial diseases and home care; in addition, only few studies examining morbidity and economic burden were available, which restricted the evaluation of the global burden for the region. To describe and compare serotype distribution across countries, the most recent available data was considered, however, in some countries the only available data was not recent and thus did not cover the same time frame. Finally, the comparability of the data reported in this review depends on several methodological aspects such as study settings, period of observation, inclusion criteria for children, and the definition of nosocomial, which vary from study to study. Research is this area is ongoing [81-85] and reflects constant interest in assessing the RVGE burden in Western Europe in countries with limited access to vaccination due to low coverage rates or absence of country specific recommendations for vaccination.

## Conclusion

In conclusion, RVGE is a common disease in Western Europe affecting the pediatric population. Data on the burden of RVGE in terms of mortality and home care is very limited for this region and while data on nosocomial infection is available variations in study setting and design may affect comparability of data. While 95% of cases are due to the main five genotypes, analysis of the evolution of different serotypes over time shows that the dominance of a certain serotype can change dramatically from year to year and from country to country. Vaccination programs may help to reduce the infection rates of this disease; a vaccine with broad and consistent serotype coverage would be important to help decrease the burden of RVGE in Western Europe.

## Competing interests

IO, HK, and MMG declare that they have no competing interests. AEK is an employee of Merck Sharp & Dohme Corp. and potentially owns stock and/or holds stock options in the Company. CG received consultancy honorarium and research grants from SPMSD, Merck and GSK-Bio.

## Authors' contributions

IO & HK developed the search algorithm and drafted the manuscript. MMG, AEK, and CG participated in the design of the methodology and drafting of the manuscript. All authors read and approved the final manuscript.

## Pre-publication history

The pre-publication history for this paper can be accessed here:

http://www.biomedcentral.com/1471-2334/12/62/prepub

## Supplementary Material

Additional file 1 **Literature capture and data sources **[15-20,23,25-34,36-58,60-66,68,71,73-77,86-106]. Click here for file
