# Supplementary material for: Burden of community-acquired and nosocomial rotavirus gastroenteritis in the pediatric population of Western Europe: a scoping review
Source: BMC Infect Dis. 2012 Mar 19;12:62. doi: 10.1186/1471-2334-12-62 (PMC3342230; doi:10.1186/1471-2334-12-62)
Supplement: Additional file 1 — Literature capture and data sources [15-20,23,25-34,36-58,60-66,68,71,73-77,86-106]. [file 1471-2334-12-62-S1.DOC]

Appendix A: Literature capture and data sources

| **Country** | **Study** | **Study setting/Population description** | **Epidemiology** | **Genotype combination** | **Morbidity and Mortality** | **Disease Burden** |
| --- | --- | --- | --- | --- | --- | --- |
|  |  |  | Incidence/ Proportion RVGE/ Seasonality |  | Disease severity/ Mortality | Resource Utilization/ Costs |
| Multicountry* | Gray, 2009[84] | EuroRotaNet surveillance database study | -/-/- | X | -/- | -/- |
| Multicountry^†^ | Iturriza-Gomara, 2009[35] | EuroRotaNet surveillance database study | -/-/X | X | -/- | -/- |
| Multicountry^‡^ | Williams, 2009[66] | Systematic review of literature | -/X/- | - | -/- | X/- |
| Multicountry^§^ | Forster, 2009^[21]^ | Prospective, multicenter study | X/X/X | X | X/- | X/- |
| Multicountry^║^ | Giaquinto, 2007[33] | Prospective, multicenter, observational study (REVEAL) | -/X/- | - | X/- | X/- |
| Multicountry^║^ | Van Damme, 2007[85] | Prospective, multicenter, observational study (REVEAL) | -/-/- | X | -/- | -/- |
| Multicountry^║^ | Van Damme, 2007[15] | Prospective, multicenter, observational study (REVEAL) | X/-/X | - | -/- | -/- |
| Multicountry^║^ | Giaquinto, 2007[64] | Costing study based on the REVEAL study | -/-/- | - | -/- | -/X |
| Multicountry^**^ | WHO, 2004[58] | 2004 WHO estimates of deaths attributable to RVGE | -/-/- | - | -/X | -/- |
| Multicountry^¶^ | Fruhwirth, 2001[14] | Three pooled prospective studies | X/-/X | X | X/- | X/- |
| Austria | Fruhwirth, 2001[27] | Prospective, multicentre study | X/X/X | - | X/- | -/- |
| Austria | Fruhwirth, 2001[75] | Economic analysis | -/-/- | - | X/- | -/X |
| Austria | Fruhwirth, 2001^[46]^ | Prospective study | -/-/- | X | -/- | -/- |
| Belgium | Bilcke, 2008[62] | Burden of illness study | -/-/- | - | X/- | X/X |
| Belgium | Rahman, 2005[86] | Prospective survey | -/-/- | X | -/- | -/- |
| Denmark | Fischer, 2007[37] | Retrospective study | -/-/X | - | -/- | X/X |
| Denmark | Fischer, 2005[47] | Prospective genotyping survey | -/-/- | X | -/- | -/- |
| Denmark | Rosenfeldt, 2005[87] | Prospective study | -/-/- | - | X/- | X/- |
| Denmark | Fischer 2001[88] | Prospective survey | -/-/X | - | -/- | X/- |
| Finland | Vesikari, 1999[31] | Retrospective database review | -/-/X | - | -/- | X/- |
| France | de Rougemont, 2009[48] | Prospective study | -/-/- | X | -/- | -/- |
| France | Fau, 2008[19] | Prospective study | X/-/X | X | X/- | X/X |
| France | Huet, 2008[16] | Prospective observational study REVEAL French results | X/X/X | X | X/X | X/- |
| France | Armengaud, 2007[42] | Retrospective hospital-based cohort study | -/X/X | - | -/- | -/- |
| France | Doit, 2007[43] | Prospective study | -/X/X | - | -/- | -/- |
| France | Marc, 2007[24] | Prospective study | X/X/- | - | -/- | X/- |
| France | Floret, 2006[20] | Prospective study | X/-/X | - | -/- | X/X |
| France | Branger, 2005[89] | One-day prevalence survey | -/X/- | - | -/- | -/- |
| France | Le Roux, 2004[90] | Prospective study | -/X/X | - | -/- | -/X |
| France | Thuret, 2004[22] | Prospective multicentre observational study | X/X/X | - | X/X | X/- |
| France | Sermet-Gaudelus, 2004[78] | Prospective multicentre observational study | -/-/- | - | -/- | X/X |
| France | Fourquet, 2003[61] | Retrospective study | -/X/- | - | X/- | X/- |
| France | Piednoir, 2003[23] | Economic burden study | X/X/X | - | -/- | X/X |
| France | Martinot, 2001[59] | Prospective multicenter study | -/-/- | - | X/- | X/- |
| France | Maille, 2000[41] | Prospective study | -/-/X | - | -/- | X/- |
| France | Rouget, 2000[39] | Prospective study | -/X/- | - | X/- | X/- |
| France | Pina, 2000[40] | Prospective study | -/X/- | - | X/- | X/X |
| Germany | Karsten, 2009[17] | Prospective multicenter study | X/-/X | - | -/- | -/- |
| Germany | Forster, 2007[91] | Retrospective record review and literature review | -/-/- | - | -/X | -/- |
| Germany | Oh, 2003[92] | Prospective survey | -/X/- | X | -/- | -/- |
| Germany | Berner, 1999[44] | Retrospective chart review | -/X/X | - | -/X | X/- |
| Greece | Kavaliotis, 2008[38] | Prospective multi-center, observational study | -/X/X | - | X/- | X/- |
| Greece | Kafetzis, 2001[93] | Prospective study | -/X/X | - | -/- | -/- |
| Ireland | Harrington, 2003[57] | Prospective observational study | -/-/- | - | X/- | X/X |
| Ireland | Lynch, 2001[36] | Retrospective database study | -/-/X | - | -/- | X/- |
| Italy | De Donno, 2009[49] | Prospective study | -/-/- | X | -/- | -/- |
| Italy | Panatto, 2009[63] | Burden of disease study | -/-/- | - | X/- | X/X |
| Italy | Palumbo, 2009[94] | Retrospective study | -/X/- | - | -/- | -/- |
| Italy | Ansaldi, 2008[50] | Community-based prospective surveillance | -/X/X | X | X/- | -/- |
| Italy | Giaquinto, 2008[18] | Prospective observational study REVEAL Italian results | X/X/X | X | X/- | X/- |
| Italy | Grassi, 2008[95] | Prospective study | -/X/X | - | -/- | -/- |
| Italy | Gabutti, 2007[96] | Retrospective study | -/X/- | - | -/- | X/- |
| Italy | Bierman, 2006[28] | Retrospective epidemiological study, | X/X/X | - | -/- | X/X |
| Italy | Arista, 2004[54] | Genotyping study | -/-/- | X | -/- | -/- |
| Italy | Martella, 2003[55] | Prospective genotyping study | -/-/- | X | -/- | -/- |
| Netherlands | Koopmans, 1999[97] | Retrospective study | -/-/X | - | -/- | -/- |
| Norway | Vainio, 2009[98] | Prospective hospital-based surveillance study | -/X/- | X | -/- | -/- |
| Norway | Flem, 2009[99] | Retrospective/prospective study | -/X/X | - | -/- | -/- |
| Portugal | Antunes, 2009[100] | Prospective multicenter study | -/X/- | X | -/- | -/- |
| Portugal | Rodrigues, 2007[101] | Prospective survey | -/X/X | X | -/- | -/- |
| Spain | Gutierrez-Gimeno, 2009[26] | Prospective observational, multicenter study | X/X/X | X | -/- | X/X |
| Spain | Gil-Prieto, 2009[25] | Retrospective study | X/X/X | - | -/X | X/X |
| Spain | Herruzo, 2009[29] | Case-control study | X/X/X | - | -/- | -/- |
| Spain | Lopez-de-Andres, 2008[80] | Retrospective study | -/X/- | - | -/X | X/X |
| Spain | Luquero Alcalde, 2008[81] | Retrospective observational study | -/-/- | - | -/- | X/X |
| Spain | Sanchez-Fauquier, 2006[102] | Prospective study | -/X/- | X | -/- | -/- |
| Spain | Gil de Miguel, 2006[103] | Retrospective study | -/-/X | - | -/- | X/- |
| Spain | Gil, 2004[82] | Retrospective study | -/-/X | - | -/- | X/X |
| Spain | Villena, 2003[51] | Prospective genotyping study | -/-/- | X | -/- | -/- |
| Spain | Visser, 1999[104] | Retrospective study | -/X/X | - | -/- | -/- |
| Sweden | Johansen, 2008[45] | Retrospective observational study | -/-/- | - | X/- | -/- |
| UK | Iturriza-Gomara, 2009[52] | Prospective observational study | -/X/X | X | -/- | -/- |
| UK | Iturriza-Gomara, 2008[53] | Prospective surveillance | -/X/- | X | -/- | -/- |
| UK | Lorgelly, 2008[83] | Cost-of-illness study | -/-/- | - | -/- | X/X |
| UK | Jit, 2007[105] | Retrospective database study | -/-/- | - | -/X | X/- |
| UK | Riordan, 2004[106] | Prospective study | -/X/- | - | -/- | -/- |
| X= data is available from study; - = data is not available from study | | | | | | |
